# Challenges in lifestyle and community interventions research; a call for innovation

**DOI:** 10.1186/s40608-014-0029-x

**Published:** 2014-12-21

**Authors:** Tommy LS Visscher, Colin Bell, Jessica S Gubbels, Terry TK Huang, Maria J Bryant, Anna Peeters, Genevieve Horne, Simone A French

**Affiliations:** Research Centre for the Prevention of Overweight Zwolle, Windesheim University of Applied Sciences and VU University, PO Box 10090, 8000GB Zwolle, The Netherlands; School of Medicine and WHO Collaborating Centre for Obesity Prevention, Deakin University, Locked Bag 20000, 3220 Geelong, VIC Australia; Department of Health Promotion, NUTRIM School for Nutrition, Toxicology and Metabolism, Maastricht University, Maastricht, The Netherlands; School of Public Health, City University of New York, New York, USA; Leeds Institute of Clinical Trials Research, University of Leeds, Leeds, LS2 9JT UK; Obesity and Population Health, Baker IDI Heart and Diabetes Institute, Melbourne, VIC Australia; Previous: BMC Obesity in-house editorial contact, BioMed Central, 236 Gray’s Inn Road, London, UK; Current address: Cancer Research UK, 407 St John Street, EC1V 4AD London, UK; Division of Epidemiology and Community Health, School of Public Health, University of Minnesota, Minneapolis, MN USA

## Abstract

Earlier this year the BMC portfolio was enriched by a new journal BMC Obesity. Here, we present the aims and objectives of the section on Lifestyle and Community Interventions. Innovative research is needed. Preventing or managing obesity requires addressing different determinants across multiple levels where diverse levers and stakeholders can play a critical role. Interactions of these determinants within and between systems need to be studied. How to leverage, manage and measure this complexity underlies the innovation that is needed in the next generation of obesity interventions. The ambition of the Lifestyle and Community Interventions section is to provide a space for innovative research, including research that falls outside the traditional comfort zone. We welcome studies of heterogeneous designs, including those of qualitative, quantitative, mixed and systems methodologies. Studies of interest include not only outcomes research of interventions but also process evaluation, cost-effectiveness or cost-benefit analysis, and implementation and dissemination research. Innovations that integrate diverse intervention levers or combine primary and secondary levels of prevention are particularly encouraged. The general aim of BMC Obesity’s Lifestyle and Community Interventions section is to advance our ability to decide on what combinations of approaches will be required to effectively and equitably prevent obesity.

## Introduction

Earlier this year the BMC series portfolio expanded to include a new journal dedicated to obesity. The five main sections of BMC Obesity are; 1) Basic science (physiology, genetics, phenotyping and metabolism); 2) Epidemiology and ethnicity; 3) Lifestyle and community interventions; 4) Policies, socioeconomic aspects, and health systems research; and 5) Treatment of obesity in clinical practice [1]. This editorial captures the ambitions and directions of the journal’s section on Lifestyle and Community Interventions as a guide to how space will be allocated in this popular and rapidly evolving field.

### Theoretical and conceptual models

As published in the launch editorial [1], the section on lifestyle and community interventions invites research on the evaluation of the outcomes, process, implementation and dissemination of community-based interventions. Although the editorial board is aware that data are crucially important to improve our understanding of integrated approaches in lifestyle and community interventions, we welcome theoretical and conceptual ideas when thoughts expressed in such papers are likely to bring our field of research forward. Hence, developing logic models and conceptual models often requires a fine balance between the data and practice-based rationale. Studies that present integrated approaches and utilize quantitative, qualitative, mixed and systems methodologies are all welcome.

#### Outcomes evaluation

Obesity prevention programs demand high-quality evaluations [2]. Given the complex nature of community interventions, it is often necessary to think more creatively when designing evaluations, which do not compromise the flexibility of the approach. Attention to design issues will ultimately lead to more successful, cost-effective randomized trials, and more rapid movement toward efficacious and effective obesity prevention programs [3]. In addition to traditional, controlled approaches to evaluation, we might need to consider alternative and innovative approaches to describe and assess ongoing real life and health promotion and obesity prevention interventions such as quasi-experimental and adaptive designs. With a number of community and lifestyle interventions now being rolled out, innovative thinking around evaluation of natural experiments will be critical. With this in mind, the journal’s section on lifestyle and community interventions welcomes papers that discuss or utilise innovative approaches in evaluation, including designs studying adaptive, and specific, measurable, attainable realistic and time-bound (SMART) elements [4]. Such designs permit the evaluation of creative and sometimes ‘changing’ interventions while maintaining the integrity of the evaluation process and ensuring minimal bias. In addition to effectiveness, the section also encourages evaluations of costs and co-benefits of interventions tested.

#### Process evaluation

Although research outputs generally focus on efficacy or effectiveness studies, there is a lot to learn from process evaluation. With the increasing scale of community-based interventions, there are new opportunities to study process elements in relation to obesity and related outcomes. On one hand, process measures such as the fidelity and dose of an intervention are key to qualifying the significance and non-significance of results. On the other hand, what we would sometimes call ‘process measures’ should be treated as outcome measures in their own right (e.g., changes in social norms), for they help us understand how interventions affect the broader context in which obesity occurs.

#### Implementation and dissemination research

Obesity prevention interventions are often dismissed because the intervention effect does not appear to last or because they have not been implemented on a large enough scale. Limitations of sustainability and scale are increasingly being addressed through implementation and dissemination research. Implementation research can, for example, include innovations on how to engage community stakeholders to improve outcomes or new strategies to enhance the adoption of evidence-based practices. Implementation research can also delve into understanding the factors that are critical to the sustainability of interventions. Tools such as the Intervention Mapping protocol provide guidelines on how to increase intervention success by appropriate applications of interventions [5]. In addition to such tools, there may also be alternative, novel strategies employed in other disciplines such as design and engineering which can improve the internal performance of interventions.

Dissemination research deals with the scale-up and diffusion of interventions across different contexts. Drivers of scale-up and diffusion may be very different than those that lead to effectiveness in the first instance. Dissemination research marks the final phase of the translational research process where proof-of-concept, efficacy and effectiveness have been demonstrated and optimized but where questions of replicability and scale remain.

The section warmly welcomes papers on implementation and dissemination issues, papers elucidating what is meant by implementation and dissemination, and how, where and why implementation and dissemination work. As a lot of expertise regarding implementation and dissemination is present amongst authors having their main interest outside the obesity research area [6], such authors are encouraged to apply their research to obesity or obesity-related issues in the spirit of trans-disciplinarity.

#### Trans-disciplinary approach

Developing solutions to the complex problem of obesity requires a trans-disciplinary and multi-sectoral approach. Innovation in designing and testing strategies that involve researchers and practitioners from diverse fields, particularly those from sectors outside of health, provide opportunities for new trans-disciplinary research approaches [7]. We aim to become a venue for research papers from diverse fields. Papers to be welcomed could well address the trans-disciplinary collaboration itself, even if such research has no direct data on obesity, as long as it is clear how the approach discussed can be used for or interpreted in the context of obesity interventions and studies.

#### Systems approach

In the last decade, obesity researchers have been paying attention not only to the different levels of influence in an ecological model but also the interactions among individuals, between individuals and their environment, and among different environmental determinants (e.g., Foresight map [8] and the ANGELO framework [9]). Kremers has elegantly combined the Theory of Planned Behaviour with the ANGELO framework, hypothesizing that cognitive and environmental determinants are very likely to interact [10]. Furthermore, in line with an ecological view on behaviour, interactions are described within the environment, between environmental types (i.e. physical, social, economic and political environment) as well as between environmental levels (i.e. the micro and macro level). In addition, interactions are described between environmental settings (e.g. between the school environment and the home environment in influencing children’s behaviour). However, such interventions need to be theorized and assessed in empirical studies [11]. Recent frameworks describe the Behavioural Change Wheel [12] and the Behavioural Change Ball [13] in which elements are addressed that are important when combating obesity in an integrated and inter-sectoral setting. We need to learn more about how different determinants interact in systems and how changes in one system affect positive or negative changes in the other systems. Furthermore, we need to learn more about how these interactions can be taken into account in preventive efforts.

In addition to better addressing interactions within and across systems, a systems perspective also acknowledges that the causal pathways contributing to obesity are dynamic and rarely linear. Given this, innovations are needed to better understand the appropriate measurements tied to a particular time scale, how to optimize the combination and sequencing of intervention strategies, and how to design interventions from a life course perspective. Systems thinking and systems methodologies (both qualitative and quantitative) offer tools for prevention and treatment that can manage such complexity and yield sustainable change.

A systems approach expands upon socio-ecological models by further emphasizing the interconnections and feedback loops among actors, factors, sectors, and levels. From a systems perspective, understanding and explicitly intervening on the interconnections and feedback loops may be important in driving the systemic changes required to normalise healthy eating and physical activity [14]. It is important to recognize that the so-called systems do not necessarily always have an obviously direct role on obesity. Further, practitioners and policy makers can benefit from reporting what does not work [14]. Papers of interest regarding systems thinking can be both hypothesis generating (e.g., modelling) or hypothesis testing (e.g., translating a systems concept into practice and testing it). The journal has a particular interest in serving as a forum for new methods to evaluate systems approaches.

#### Linking weight gain prevention and weight management

Although prevention has been the primary concern of the health promotion field, it is increasingly recognized that to significantly reduce the overall prevalence of obesity, treatment is also required. To date, little has been done at scale to implement prevention and treatment strategies simultaneously. Professionals from the weight management domain often lack competencies needed to promote health behaviour [15]. The challenge is to organize health promotion and health care appropriately, in which for example expertise that is available in the prevention area can be applied in the management area. The journal welcomes innovative thinking and studies linking prevention and management, from new intervention designs to how new clinical and public health informatics are integrated to enhance prevention and care. Papers on organizational and economic issues are also welcome, including the issue on who pays for obesity-related costs.

#### Innovations in obesity research

Innovations are needed at the conceptual, methodological and tactical levels to combat the obesity epidemic worldwide. True innovation is disruptive and may well take place beyond the scope of our own context and comfort zone. To move the field of obesity prevention and management forward, there is a need to embrace this discomfort. We need to learn from other disciplines, other sectors and other systems than the ones in which we are used to working. The section of Lifestyle and Community Intervention aims to offer a home to bold and disruptive ideas.

## Conclusion

Let the challenge be about moving forward in obesity prevention and management. As Rutter said, ‘The challenge is huge, but the risks of failures are greater [16].” Without calling for unthoughtful or methodologically flawed studies, any prospective author might envision being referred to in 25 years’ time as “the author who published that innovative approach in that new journal BMC Obesity when no other journal dared”.
